# Biochemical and structural characterization of the interface mediating interaction between the influenza A virus non-structural protein-1 and a monoclonal antibody

**DOI:** 10.1038/srep33382

**Published:** 2016-09-16

**Authors:** Jianping Wu, Chee-Keng Mok, Vincent Tak Kwong Chow, Y. Adam Yuan, Yee-Joo Tan

**Affiliations:** 1 Department of Microbiology and Immunology, Yong Loo Lin School of Medicine, National University Health System (NUHS), National University of Singapore, Singapore; 2 Department of Biological Sciences, Faculty of Science, National University of Singapore, Singapore; 3 National University of Singapore (Suzhou) Research Institute, Suzhou Industrial Park, Jiangsu 215123, China; 4 Institute of Molecular and Cell Biology, A*STAR (Agency for Science, Technology and Research), Singapore

## Abstract

We have previously shown that a non-structural protein 1 (NS1)-binding monoclonal antibody, termed as 2H6, can significantly reduce influenza A virus (IAV) replication when expressed intracellularly. In this study, we further showed that 2H6 binds stronger to the NS1 of H5N1 than A/Puerto Rico/8/1934(H1N1) because of an amino acid difference at residue 48. A crystal structure of 2H6 fragment antigen-binding (Fab) has also been solved and docked onto the NS1 structure to reveal the contacts between specific residues at the interface of antibody-antigen complex. In one of the models, the predicted molecular contacts between residues in NS1 and 2H6-Fab correlate well with biochemical results. Taken together, residues N48 and T49 in H5N1 NS1 act cooperatively to maintain a strong interaction with mAb 2H6 by forming hydrogen bonds with residues found in the heavy chain of the antibody. Interestingly, the pandemic H1N1-2009 and the majority of seasonal H3N2 circulating in humans since 1968 has N48 in NS1, suggesting that mAb 2H6 could bind to most of the currently circulating seasonal influenza A virus strains. Consistent with the involvement of residue T49, which is well-conserved, in RNA binding, mAb 2H6 was also found to inhibit the interaction between NS1 and double-stranded RNA.

Influenza A viruses (IAVs) constantly circulate in animal hosts including birds, human and pigs. Seasonal IAVs are one of the major causes of respiratory tract infections and responsible for 3–5 million clinical infections and 250,000–500,000 fatal cases annually^1^. IAV is a negative sense single-stranded RNA virus with segmented genomes^2^, which belongs to the family *Orthomyxoviridae* and is subtyped based on its surface glycoproteins haemagglutinin (HA) and neuraminidase (NA). So far, 18 HA and 11 NA subtypes have been identified^3^, with the H1N1 and H3N2 subtypes being the seasonal IAVs currently circulating in human^4^.

Currently, vaccination is still considered the first line of defence against influenza viral infection^5^, however it needs to be reformulated annually due to the genetic variability of the virus^6^. The conventional influenza vaccine aims to stimulate immunity to produce antibodies against the viral envelope HA protein. Unfortunately, these antibodies are mainly strain specific, in which case IAV might be able to evade the recognition of the antibody by constantly mutating the antigenic determinants^7^. Thus, one way to overcome this limitation is to produce and/or engineer antibodies that could neutralize most viral strains. Alternatively, another option to combat IAV is the use of antiviral compounds, which include two classes of drugs. One is directed against M2 ion channel protein to block the uncoating of virus after its entry into the host cells^8^ and another is against NA to block the release of newly formed virions to surrounding uninfected cells^9^. As resistance to these two classes of antiviral drugs has occurred in the circulating strains of the IAVs^10^, there is an urgent need to develop new therapeutic approaches.

Non-structural protein 1 (NS1) of IAV is a potent type I interferon (IFN) antagonist, although the mechanism of inhibiting the IFN response is strain dependent^11^. NS1 typically contains 230 amino acid residues (~26 kDa), although there are variations among various subtypes and strains^12^. NS1 has two functional domains, namely the N-terminal RNA binding domain (RBD) and C-terminal effector domain (ED), connected by a flexible linker^13^.

One of the most striking features of NS1 is its ability to bind to different species of RNA including double-stranded RNA (dsRNA), viral RNA (vRNA), 3′ poly-A tail of mRNAs and small nuclear RNAs (snRNA)^14^,^15^,^16^ via its RBD. By binding to and sequestering dsRNA from 2′–5′ oligo (A) synthetase (OAS)/RNase L pathway, NS1 protects IAV against the antiviral state induced by IFN-β^17^. NS1 could also inhibit ubiquitin ligase activity of Tripartite motif-containing protein 25 (TRIM25) to modulate retinoic acid-inducible gene I (RIG-I) induced IFN response^18^. Recently, the direct interaction between RIG-I and NS1 with strain specificity has been reported^19^, which provided the structural basis for how this interaction might modulate virulence during the infection. Besides, direct binding of NS1 to protein kinase R (PKR) could help IAVs counteract PKR-mediated anti-viral response^20^. NS1 has also been shown to interact directly with the p85β regulatory subunit of phosphoinositide 3-kinase (PI3K) but it is unclear how this interaction contributes to apoptosis regulation in infected cells^21^,^22^.

Given the multifunctional properties of the NS1 protein, much effort has been directed towards the development of NS1-based antiviral strategy^23^,^24^. For example, numerous novel inhibitors targeting NS1 proteins have been identified and demonstrated significant antiviral activities *in vitro*^25^,^26^,^27^. In our previous study, we used full-length NS1 protein of H5N1 IAV to generate a monoclonal antibody (mAb) 2H6 and found that it could cross-react with NS1 of H3N2 and H1N1 subtypes^28^. MAb 2H6 binds to the RBD of NS1(RBD) and the intracellular expression of 2H6-single-chain variable fragment (scFv) in mammalian cells reduced the replication of A/Puerto Rico/8/1934(H1N1) (H1N1-PR8) virus^29^. In the present study, we used biochemical, structural and modelling methods to define the molecular interface mediating the interaction between mAb 2H6 and NS1. An AlphaScreen assay was also set up to determine if mAb 2H6 could interfere with the interaction of NS1 with dsRNA.

## Results

### Residues 30–53 in H5N1-NS1 are sufficient for its interaction with mAb 2H6

**Figure 1:**
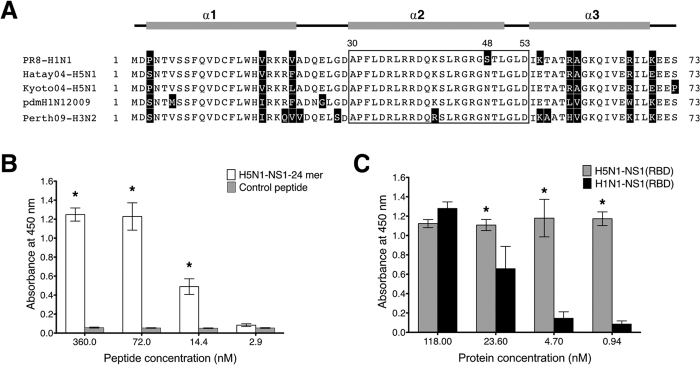
Binding region of mAb 2H6 within NS1(RBD). (**A**) Sequence alignment of NS1(RBD) of H1N1 (A/Puerto Rico/8/1934), H5N1 (A/Hatay/2004), H5N1 (A/crow/Kyoto/T1/2004), pandemic H1N1 (pdmH1N1) (A/Canada/GFA0402/2009) and H3N2 (A/Perth/16/2009). Mismatches are shown in white letters. NS1(RBD) is composed of 3 α-helices as shown above the sequence. The region corresponding to helix α2 (residues 30–53) is boxed. (**B**) Peptide ELISA was performed to determine the region of NS1 sufficient for binding to mAb 2H6. Wells were coated with serially diluted H5N1-NS1-24mer or a negative control peptide and probed with 5 μg/ml mAb 2H6. ^*^Indicates statistically significant difference of *p *< 0. 05 when compared with control peptide. (**C**) Comparative ELISA was performed to determine the ability of NS1(RBD) of H1N1-PR8 and H5N1 to bind to mAb 2H6. Wells were coated with serially diluted proteins and probed with 5 μg/ml mAb 2H6. Data shown represents result from three independent experiments and error bars represent standard deviation (SD) of the experiment carried out in duplicates. *Indicates statistically significant difference of *p* < 0.05 when compared with H1N1-PR8-NS1(RBD).

As described previously^28^, the deletion of residues 42–53 of NS1 abolished its interaction with mAb 2H6. These residues lie in the helix α2 (residues 30–53) of H5N1-NS1(RBD) and are well conserved between H5N1, H1N1 and H3N2 viruses (Fig. 1A). This is consistent with the ability of mAb 2H6 to bind to both avian H5N1 and seasonal IAVs^28^. In order to determine if the helix α2 of H5N1-NS1(RBD) is sufficient for the interaction with mAb 2H6, enzyme-linked immunosorbent assay (ELISA) was performed by using a synthetic peptide H5N1-NS1-24mer corresponding to helix α2. As shown in Fig. 1B, mAb 2H6 bound to H5N1-NS1-24mer in a dose dependent manner, indicating that the helix α2 of NS1(RBD) is sufficient for its interaction with mAb 2H6. In contrast, there was no binding between mAb 2H6 and an irrelevant control peptide of similar molecular weight.

Within helix α2, there is only one amino acid difference between H5N1 and H1N1-PR8, namely N48 in H5N1-NS1 and S48 in H1N1-PR8-NS1 (Fig. 1A). To determine if residue 48 in NS1 is involved in its interaction with mAb 2H6, recombinant H5N1-NS1(RBD) and H1N1-PR8-NS1(RBD) proteins were bacterially expressed and purified for ELISA. The ELISA readings were similar for H5N1-NS1(RBD) and H1N1-PR8-NS1(RBD) when high concentrations of proteins were coated on the plate (Fig. 1C). However, the readings were significantly higher for H5N1-NS1(RBD) at lower protein concentrations, indicating that that the single amino acid difference between helix α2 of H5N1-NS1(RBD) and H1N1-PR8-NS1(RBD) affects their interactions with mAb 2H6 (Fig. 1C).

### Residue 48 in NS1 is critical for its interaction with mAb 2H6

**Figure 2:**
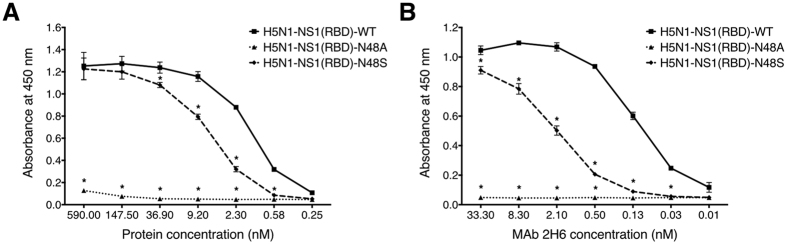
Determination of the effect of single amino acid substitution at residue 48 in NS1(RBD) for the interaction with mAb 2H6. (**A**) Wells were coated with different concentration of H5N1-NS1(RBD)-WT (full line), H5N1-NS1(RBD)-N48A (dashed line) and H5N1-NS1(RBD)-N48S (dotted line) and probed with 5 μg/ml of mAb 2H6. (**B**) 125 μg/ml of WT (full line), N48A (dashed line) and N48S (dotted line) proteins were probed with decreasing concentration of mAb 2H6. All experiments were performed in triplicates, and the average values with SD are plotted. *Indicates statistically significant difference of *p* < 0.05 when compared to H5N1-NS1(RBD)-WT.

To further define the contribution of residue 48 in H5N1-NS1 to the interaction with mAb 2H6, two mutant proteins in which N was mutated to A and S respectively, were generated. Comparative ELISA showed that mAb 2H6 bound to NS1(RBD)-wild-type (WT) stronger than NS1(RBD)-N48S when different amounts of protein were coated on the plate and analyzed with 5 μg/ml of mAb 2H6 (Fig. 2A). Similarly, when a fixed amount of protein (125 μg/ml) was coated on the plate and analyzed with different concentrations of mAb 2H6, the binding to NS1(RBD)-N48S was significantly lower than NS1(RBD)-WT (Fig. 2B). Furthermore, when N was substituted with A at residue 48, the interaction between mAb 2H6 and NS1(RBD) was totally abolished in all the conditions tested (Fig. 2A,B). This result suggests that the difference at residue 48 is the main reason for the stronger binding of mAb 2H6 to H5N1-NS1(RBD) when compared to H1N1-PR8-NS1(RBD) (Fig. 1). Since both N48 and S48 are polar amino acids while A48 has no side chain, it is probable that the formation of hydrogen bonds is important for the interaction between mAb 2H6 and NS1.

### MAb 2H6 binds differently to H1N1-PR8 virus when compared with mutant viruses carrying substitution at residue 48 in NS1

**Figure 3:**
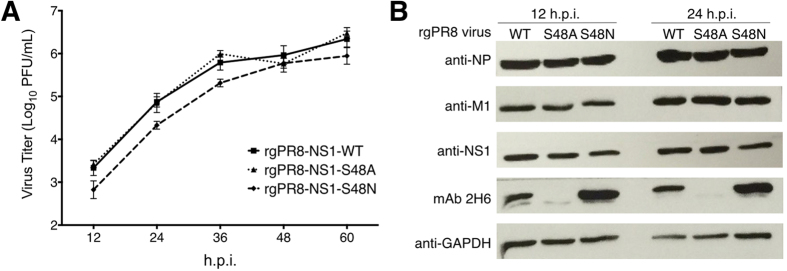
Binding of mAb 2H6 to rgPR8 WT and mutant viruses carrying substitution at residue 48 of NS1. (**A**) Multi-cycle growth kinetics of WT and mutant viruses were obtained by infecting A549 cells with rgPR8-NS1-WT (full line), rgPR8-NS1-S48A (dashed line) or rgPR8-NS1-S48N (dotted line) viruses at MOI of 0.01. Virus production at different time points post infection was determined using plaque assay. (**B**) 293T cells with rgPR8-NS1-WT, rgPR8-NS1-S48A or rgPR8-NS1-S48N mutant viruses at MOI of 2. Cell lysates were collected at 12 or 24 h.p.i. and subjected to Western blot analysis. The experiments were repeated three times and a representative set of data is shown.

Since mAb 2H6 binds to bacterially-expressed NS1 protein of H5N1 and H1N1-PR8 with different affinities, it is important to investigate whether this holds true for NS1 expressed in infected cells. Thus, recombinant PR8 (rgPR8) viruses expressing the NS1 protein containing a single amino acid substitution at residue 48 (rgPR8-NS1-S48A and rgPR8-NS1-S48N) were generated using a reverse genetics system. Subsequently, A549 cells were infected with each virus at a low multiplicity of infection (MOI) of 0.01 respectively and plaque assay was used to determine the amount of virus secreted at different time points post-infection. As shown in Fig. 3A, although the viral titer was slightly lower in the case of rgPR8-NS1-S48N infection, the overall growth rates of WT and mutant viruses were similar from 12 to 60 hours post-infection (h.p.i.). This is consistent with a previous report showing that substitution of residue 48 in NS1 does not affect viral replication *in vitro*^30^.

Next, 293T cells were infected with 2 MOI of viruses and cell lysates were collected at 12 and 24 h.p.i. to determine the rate of viral protein synthesis. Consistent with the virus growth kinetics, the expressions of structural proteins NP and M1 were similar for all 3 viruses at both time-points (Fig. 3B). The level of NS1, as determined by using a rabbit anti-NS1 polyclonal antibody, was also comparable for all 3 viruses. However, mAb 2H6 bound to rgPR8-NS1-S48N virus significantly stronger than rgPR8-NS1-WT virus containing S48 residue in NS1, supporting the above results that N48 in NS1 is preferred over S48 for the interaction with mAb 2H6. As expected, mAb 2H6 did not bind to rgPR8-NS1-S48A virus.

### HADDOCK-derived model of the complex between 2H6-fragment antigen-binding (Fab) and NS1(RBD)

To further define the interaction interface between mAb 2H6 and NS1, attempts were made to co-crystallize the complex but failed. However, the 2H6-Fab alone was found to crystallize in space group P2(1) and the structure was solved by molecular replacement using the structure of BL3-6 (PDBID: 4Q9Q) as the starting model and refined to the crystallographic *R*-factor of 28.5% (deposited in the PDB under accession code 5B4M). The refinement statistics are shown in Table 1.

**Figure 4:**
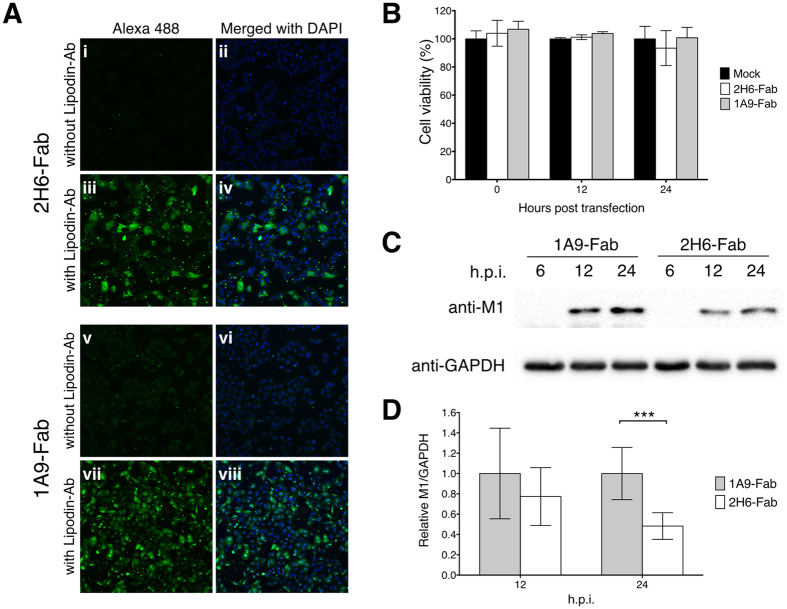
Effect of 2H6-Fab on the replication of influenza A virus. (**A**) Efficiency of Fab delivery into A549 cells via Lipodin-Ab transfection was determined by immunofluorescence assay performed with an Alexa Fluor 488-conjugated goat anti-mouse IgG antibody (green). Nuclei were counterstained with DAPI (blue). Cells were treated with 2H6-Fab (i & ii) or 1A9-Fab (v & vi) alone or 2H6-Fab with Lipodin-Ab (iii & iv) or 1A9-Fab with Lipodin-Ab reagent (vii & viii) for 12 h, washed with PBS and then cultured in serum-free MEM medium for another 24 h, followed by immunofluorescence assay. (**B**) A549 cells transfected with 2H6-Fab or 1A9-Fab were analyzed for cell viability at 0, 12, 24 hours post transfection. The percentage of cell viability of mock transfected samples at each time point was arbitrarily set to 100%. The percentage of the other samples relative to mock transfected samples was then determined. (**C**) A549 cells transfected with 2H6-Fab or 1A9-Fab were infected by rgPR8-NS1-S48N virus (MOI of 1) and harvested at 6, 12, 24 h.p.i. Western blot analysis was performed by using anti-M1 and anti-GAPDH antibodies. (**D**) The expression levels of M1 were normalized to GAPDH and the value for 1A9-Fab at each time-point was arbitrarily set to 1. All experiments were performed in triplicates, and the average values with SD are plotted. Differences in M1 expression between 2H6-Fab and 1A9-Fab transfected cells were evaluated by unpaired t-test (***p < 0.001).

Previously, we demonstrated that the intracellular expression of 2H6-scFv in mammalian cells reduced the replication of PR8 virus. Since the three dimensional structure of 2H6-Fab has been solved, a commercially available Lipodin-Ab reagent (Abbiotec), which is a protein transfection reagent dedicated to the transport of antibodies into living cells, was used to deliver 2H6-Fab into A549 cells. The cells were then subjected to infection so as to determine if 2H6-Fab has an impact on viral replication. When either 2H6-Fab or 1A9-Fab (which is derived from a negative-control antibody binding to the spike glycoprotein of SARS coronavirus)^31^ was transfected into A549 cells using Lipodin-Ab reagent, intracellular accumulation of Fab was observed even up to 24 h post transfection (Fig. 4A). The intracellular accumulation of Fab was also observed at as early as 4 h post transfection (data not shown). The transfection efficiency was about 70% and most of the Fab molecules were evenly distributed in the cytoplasm but there were some punctate staining which could be due to aggregation of Fab inside A549 cells. In contrast, no intracellular accumulation of Fab was observed when 1A9-Fab or 2H6-Fab was added to the cells without Lipodin-Ab reagent. As shown in Fig. 4B, the delivery of Fab did not have any significant effect on cell viability at either 12 or 24 h post transfection.

Upon successful delivery of Fab, its biological function was assessed in influenza A virus infected cells. As mAb 2H6 binds strongly to rgPR8-NS1-S48N virus (Fig. 3), A549 cells were infected with rgPR8-NS1-S48N virus after transfection of 2H6-Fab. Cell lysates were collected at 6, 12, 24 h.p.i. to determine the rate of viral protein synthesis. As shown in Fig. 4C,D, the level of viral M1 protein at 24 h.p.i. was significantly reduced in 2H6-Fab transfected cells when compared to 1A9-Fab transfected cells. At 24 h.p.i., the average reduction in normalized M1 expression in 2H6-Fab transfected cells was 50% when compared to 1A9-Fab transfected cells. At 12 h.p.i., a slight reduction in M1 expression (~25%) was also observed in 2H6-Fab transfected cells but it is not statistically significant. This result suggests that the successful delivery of 2H6-Fab into living cells could reduce viral replication by affecting certain function(s) of NS1 in the infected cells.

Next, computational modelling was used to study the complex between this high resolution 2H6-Fab structure and the published structure of H5N1-NS1(RBD) of H5N1/A/crow/Kyoto/T1/2004 strain (PDB ID: 2Z0A). This was conducted by using HADDOCK on 192 water-refined models, including an analysis of energy contributions from Van der Waals interaction, electrostatic interaction, restraints violation and buried surface area^32^. As comparative ELISA in this and previous studies^29^ showed that residues N48 and T49 in NS1(RBD) are important for the interaction with mAb 2H6, they were defined as active residues involved in the binding interaction to generate a series of models of the NS1(RBD) and 2H6-Fab complex.

**Figure 5:**
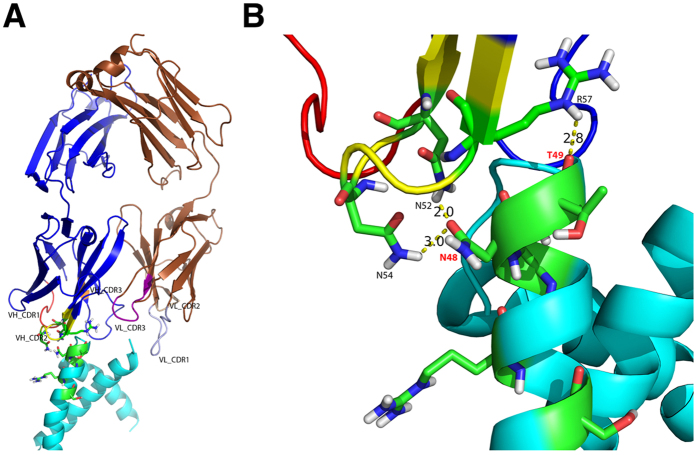
HADDOCK-derived structure model of 2H6-Fab and H5N1-NS1(RBD) complex. (**A**) Ribbon diagram of HADDOCK-derived model with NS1(RBD) coloured in cyan, 2H6-Fab heavy chain coloured in blue and light chain coloured in brown. At the antigen-antibody interface, VH-CDR1, CDR2 and CDR3 of 2H6-Fab were coloured in red, yellow and orange respectively whileVL-CDR1, CDR2 and CDR3 were coloured in grey, wheat and purple respectively. (**B**) Focused view of the binding interface displaying the residues involved in the interaction between antigen and antibody. Probable residues of NS1(RBD) and 2H6-Fab critical for the binding are shown in stick mode and numbered in red and black respectively. Probable hydrogen bonds are indicated by dashed line. All images of the models were generated with PyMOL^44^.

All the models of NS1(RBD) and 2H6-Fab complex were found to cluster into 3 groups, in which there were at least two conformations of the ensemble showing backbone root-mean-square deviations at the interface of less than 1.0 Å. As additional ELISA results showed that 3 residues, namely S42, R44, and G47, in NS1 are not involved in interaction with mAb 2H6 (Figure S1), this information was used to distinguish between the models in these 3 clusters. Of all energetically best models generated, 9 predicted models were grouped into cluster 2. The average buried surface area was 1670.7 ± 52.6 Å^2^, and RMSD from the overall lowest-energy structure was 4.9 ± 0.3 Å. Among them, the best predicted model from this cluster showed good agreement with our comparative ELISA data, since only residues N48 and T49 were predicted to be involved in the interaction, while the side chains of S42, R44 and G47 were either distal from the interface (S42 and R44) or inaccessible (G47) to the binding partner of 2H6-Fab (Fig. 5). By analyzing the polarity of the amino acids and distance between them in this model, it is predicted that residues N52 and N54 in the variable domain of heavy chain (VH)-complementarity determining region 2 (CDR2) of 2H6-Fab could form hydrogen bonds with the side chain of N48 in NS1(RBD). In addition, the side chain of T49 in NS1(RBD) could form hydrogen bonds with residue R57 in VH-CDR2. In contrast, VH-CDR1, VH-CDR3 and all the CDRs in the variable domain of light chain (VL) are unlikely to be involved in the interaction as they are distal to helix α2 of NS1(RBD).

On the other hand, 179 predicted models were grouped into cluster 1. For these refined structures analyzed, the average buried surface area was 1234.9 ± 22.5 Å^2^, and RMSD from the overall lowest-energy structure was 1.1 ± 0.8 Å. Based on the best predicted model from this cluster (Figure S2), it was predicted that T49 of NS1(RBD) could form the hydrogen bond with N52 of VH-CDR2 while N48 of NS1(RBD) could form the hydrogen bond with N96 of VL-CDR3. These predictions are in agreement with the results shown in Fig. 2 and in our previous publication^29^. However, this model also predicted that R44 of NS1(RBD) could be involved in the interaction with 2H6-Fab because it was in close proximity to two residues in VL-CDR1. In this model, the distance between R44 of NS1(RBD) and Y31 of VL-CDR1 was 2.1 Å while the distance between R44 of NS1(RBD) and S35 of VL-CDR1 was 2.9 Å. Thus, this model does not agree with the results from comparative ELISA which showed that substitution of R44 of NS1(RBD) with K did not affect its interaction with mAb 2H6 (Figure S1).

Lastly, another 4 predicted models were grouped into cluster 3. The average buried surface area was 1174.4 ± 22.1 Å^2^, and RMSD from the overall lowest-energy structure was 7.3 ± 0.6 Å. Based on the best predicted model from this cluster (Figure S2), the contacts between N48 and T49 with residues in 2H6-Fab were the same as described above for the model from cluster 1. However, this model also predicted that R44 of NS1(RBD) could be involved in the interaction with 2H6-Fab because it was in close proximity to three residues in VL-CDR1. In this model, Y31, S35 and Y36 of VL-CDR1 were found to be at 1.7 Å, 3.3 Å and 2.8 Å from R44 of NS1(RBD) respectively. Thus, this model does not agree with the results from comparative ELISA which showed that substitution of R44 of NS1(RBD) with K did not affect its interaction with mAb 2H6 (Figure S1).

Overall, the predicted model from cluster 2 is consistent with our comparative ELISA data and suggests that residues N48 and T49 are important for the binding between NS1(RBD) and 2H6-Fab because their side-chains could make hydrogen bonds with residues in the VH-CDR2 of the Fab. In addition, R44 of NS1(RBD) was distal from the antibody-antigen interface, which is consistent with the results from comparative ELISA (Figure S1) showing that substitution of R44 of NS1(RBD) with K did not affect its interaction with mAb 2H6. In contrast, the predicted models from cluster 1 and cluster 3 do not agree with the results from comparative ELISA.

### MAb 2H6 disrupts NS1 and dsRNA interaction

**Figure 6:**
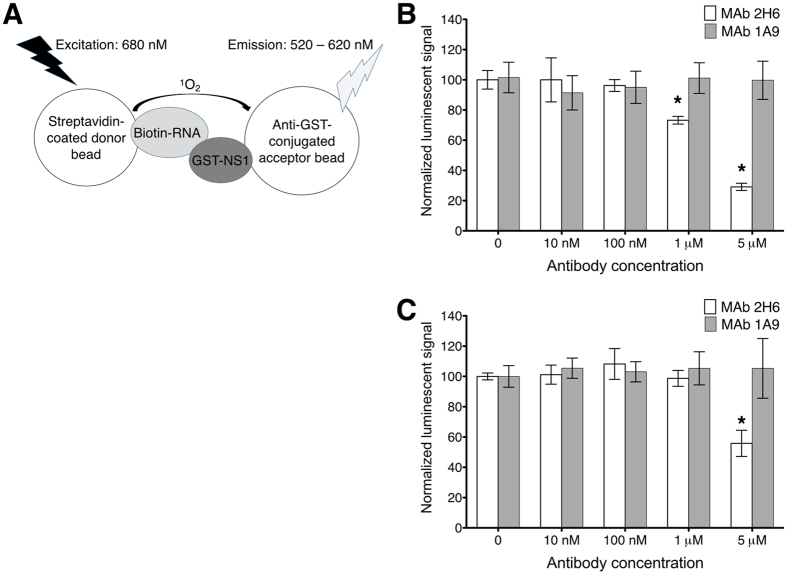
Determination of the effect of mAb 2H6 on NS1(RBD)-dsRNA interaction. (**A**) Schematic representation of an AlphaScreen assay designed to measure the interaction between NS1 and dsRNA *in vitro*. GST-tagged NS1 protein was incubated with biotinylated dsRNA and the interaction between NS1 and dsRNA could bring streptavidin-coated donor bead and anti-GST-conjugated acceptor bead close to each other. When excited at 680 nM, singlet oxygen molecules (^1^O_2_) are produced from donor beads, which react with acceptor beads to produce light emission measured at 520–620 nM. (**B**) Inhibition activity of mAb 2H6 was determined by pre-incubating 10 nM GST-tagged NS1 protein with serially diluted mAb 2H6 or a negative control mAb 1A9. Following that, luminescent signal was measured after the addition of 10 nM biotinylated dsRNA, acceptor and donor beads. (**C**) Inhibition activity of mAb 2H6 was determined by incubating 10 nM GST-tagged NS1 protein, 10 nM biotinylated-dsRNA and serially diluted mAb 2H6 or 1A9 simultaneously. Following that, luminescent signal was measured after the addition of acceptor and donor beads. All readings obtained were normalized against that of samples in the absence of antibody. Error bars represent SD of the experiment carried out in triplicates. *Indicates statistically significant difference of *p* < 0.05 when compared to mAb 1A9.

NS1(RBD) forms a symmetric six-helical homodimer, which binds to dsRNA. The key residues in NS1 involved in the interaction with dsRNA are T5, D29, D34, R35, R37, R38, L41, S42 and T49, most of which are positively charged residues and mainly clustered in the middle of helices α2/α2′ of the RBD^33^,^34^. To investigate whether mAb 2H6 hampers dsRNA-NS1 interaction *in vitro*, an AlphaScreen assay was carried out. In this experiment, glutathione S-transferease (GST)-tagged H5N1-NS1(RBD) protein, which was produced in *E. coli*, was incubated with synthetic 21-nucleotide siRNA (21nt-siRNA) followed by addition of streptavidin coated donor beads and anti-GST-conjugated acceptor beads which recognize biotinylated RNA and GST-tagged protein respectively. If the interaction between NS1 and 21nt-siRNA brings both beads to close proximity, transfer of excitation energy from donor beads into acceptor beads will yield a luminescent signal (Fig. 6A)^35^. When NS1(RBD) was pre-incubated with different concentrations of mAb 2H6 followed by addition of 21nt-siRNA, acceptor and donor beads, the luminescent signal decreased at high concentration of mAb 2H6 (Fig. 6B). The luminescent signal was reduced by ~30% and ~70% at mAb 2H6 concentrations of 1 and 5 μM respectively (Fig. 6B), suggesting that the binding of mAb 2H6 to NS1(RBD) can block the interaction of NS1 with dsRNA. On the other hand, the negative control mAb 1A9 did not reduce the luminescent signal at all the concentrations tested.

Furthermore, when mAb 2H6, NS1 and 21nt-siRNA were mixed simultaneously, 5 μM of mAb 2H6 could reduce the signal by about 30% (Fig. 6C), which suggest that mAb 2H6 also directly competes with dsRNA to bind to NS1.

## Discussion

The NS1 protein of influenza virus is a multi-functional protein that is involved in key aspects of the virus replication cycle^36^. The NS1(RBD) consists of the first 73 amino acids and contains residues critical for dsRNA binding^14^. The dimerization of the RBD is a prerequisite for its RNA binding activity^13^. Since the NS1 protein is an intracellularly expressed viral protein, which is subjected to less host selective immune response and thus has lower mutation rate, antibodies targeting this protein could be helpful for the development of therapeutic treatment of influenza A infection. Indeed, our previous study showed that mAb 2H6 binds to the highly conserved T49 residue in NS1 and reduces viral replication of H1N1-PR8 in mammalian cell lines^29^.

In this study, comparative ELISA showed that helix α2 of NS1(RBD) is sufficient for its interaction with mAb 2H6 (Fig. 1). While mAb 2H6 binds to NS1(RBD) of both H5N1 and H1N1-PR8, the binding affinity to the homologous H5N1 viral protein is higher. Interestingly, a single amino acid difference at residue 48 in helix α2 of NS1(RBD) of H5N1 and H1N1-PR8 is found to be critical for the interaction with mAb 2H6 (Figs 2 and 3). By using either purified NS1 protein or NS1 expressed in infected cells, our results showed that the interaction of mAb 2H6 with NS1 is stronger when residue 48 is N than when it is S and is abolished when the residue is an A.

To understand the pattern of polymorphism at residue 48 in NS1, sequences were retrieved from NIAID Influenza Research Database (http://www.fludb.org) and analyzed (Table 2). Sequence analysis of avian H5N1 isolates revealed that 87% of them have N48 and 13% have S48 suggesting that mAb 2H6 has the ability to bind to the majority of avian H5N1 isolates. Interestingly, a recent computational study compared the viral proteins of highly and lowly pathogenic H5 viruses and identified S48 to N substitution as a potential marker of pathogenicity of avian influenza virus subtype H5^37^. As for human isolates, the majority (93%) of seasonal H1N1 isolated before 2009 have S48 in NS1 like H1N1-PR8. However, almost all the pandemic 2009 H1N1 (pdmH1N1) isolates have N48 in NS1. As for seasonal H3N2, 91% of viruses isolated before 2009 have N48 and this percentage increased to 99% for those isolated from 2009 to 2015. Thus, mAb 2H6 is expected to bind to the majority of circulating seasonal influenza viruses.

By solving a crystal structure of 2H6-Fab and docking it onto the NS1(RBD) of H5N1 with the HADDOCK program, molecular contacts made by residues T49 and N48 at the interface of antibody-antigen complex were predicted (Fig. 5). Based on one of the energetically best models generated, the side chain of residue N48 in NS1(RBD) could form hydrogen bonds with the side chains of residues N52 and N54 of VH-CDR2 of 2H6-Fab. Meanwhile, residue T49 in NS1(RBD) seems to interact with residue R57 in VH-CDR2. These predictions are consistent with the results of binding assays performed using substitution mutants of NS1 as described above and in our previous study^29^. While the predicted 3D model of the antibody-antigen complex gives us some clues on how residues 48 and 49 in NS1(RBD) interact with 2H6-Fab, it may be able to accurately reveal the contributions of all molecular contacts between NS1(RBD) and 2H6-Fab. Hence, further studies could focus on the use of other epitope mapping methodologies^38^, besides crystallography, to obtain a more precise map of the molecular contacts at the antigen and antibody interface.

Furthermore, an AlphaScreen assay showed that mAb 2H6 could inhibit the interaction between NS1 and dsRNA (Fig. 6). While both residues 48 and 49 are located in helix α2 of the NS1(RBD), it has been shown that T49, but not S48, is one of the key residues involved in dsRNA binding^34^. Thus, the interaction between T49 and 2H6-Fab predicted in the docked model is consistent with the ability of mAb 2H6 to inhibit the interaction between NS1 and dsRNA. Although a previous study has demonstrated that mutation at residue 48 did not affect the affinity of NS1(RBD) for dsRNA^30^, our study suggests that the side chain of N48 makes crucial contacts with mAb 2H6 to stabilize the antibody-antigen complex. As such, when the side chain is not present in the case of the N48A substitution mutant, interaction with mAb 2H6 is completely abolished (Figs 2 and 3). This indicates that the interaction between the side chains of residue T49 in NS1 and residue R57 in 2H6-Fab is not sufficient to maintain the antibody-antigen interface. On the other hand, the T49V and T49A substitution mutants still retained some binding to mAb 2H6 (Figure S1), presumably because of the hydrogen bonds between residue N48 and the heavy chain of the antibody.

Based the AlphaScreen assay, a high concentration of mAb 2H6 (~5 μM) was required to reduce the interaction between NS1(RBD) and dsRNA by >50% (Fig. 6). On the other hand, the concentration of mAb 2H6 required for binding NS1(RBD) in ELISA was in the nanomolar range (Fig. 2). This discrepancy could be due to the differences between the two assays but it also suggests that the binding of mAb 2H6 to NS1 could have other effects on NS1 besides disrupting its interaction with dsRNA. Indeed, our previous gel filtration and dynamic light scattering results suggest that the complex between 2H6-Fab and NS1(RBD) is multimeric in nature and each oligomer could consist of 6 molecules of NS1(RBD) and 6 molecules of 2H6-Fab^29^. In contrast, NS1(RBD) eluted out the gel filtration column as a dimer, as would be expected, in the absence of 2H6-Fab^29^. In addition, the delivery of 2H6-Fab into A549 cells also caused a reduction in the replication of rgPR8-NS1-S48N recombinant virus (Fig. 4). While it is difficult to precisely define the biologically relevant quaternary structures of NS1, several studies have shown that the NS1 has conformational plasticity and dynamic changes in the quaternary structure of NS1 are likely to be important for the different functions of NS1 in infected cells^39^. Hence, it is possible that the binding of 2H6-Fab to the NS1 expressed in infected A549 cells could affect the conformational plasticity of NS1 and/or its ability to interact with certain cellular factor, thus resulting in a reduction in viral replication. In future studies, advanced fluorescence microscopic techniques could be used to determine the effect of 2H6-Fab on the dynamic of NS1 inside infected cells.

In summary, we have used biochemical and structural methods to characterize the interaction between NS1 and mAb 2H6. Our results showed that helix α2 in NS1(RBD) is sufficient for interacting with mAb 2H6 and residues N48 and T49 in this helix are likely to make hydrogen bonds with the CDR2 of the antibody heavy chain. Helix α2 is highly conserved and this is consistent with the ability of mAb 2H6 to bind to different subtypes of IAV. After solving a high resolution crystal structure of 2H6-Fab, a HADDOCK-derived model of the antibody-antigen complex has been obtained and the molecular contacts predicted from this model are in agreement with results obtained from comparative ELISA performed using NS1 mutants. This model may be used in antibody engineering experiments to increase the affinity of interaction between NS1 and mAb 2H6 so as to increase mAb 2H6’s potency in viral inhibition. In addition, it may be useful in structure-based rational drug design to identify small molecule inhibitors of NS1.

## Methods

### Cells

A549, 293T and MDCK cells were purchased from American Type Culture Collection (Manassas, VA, USA). A549 cells were cultured in Minimum Essential Medium (MEM) (Gibco). 293T and MDCK cells were cultured in Dulbecco’s Modified Eagle’s Medium (Invitrogen). Both media were supplemented with 10% fetal bovine serum (Hyclone), penicillin (10,000 units/ml)-streptomycin (10 mg/ml) solution (Sigma Aldrich). All cell lines were maintained at 37 °C with 5% CO_2_.

### Ascites and rabbit polyclonal antibodies production

Ascites were produced by injecting hybridoma cells into peritoneal cavities of pristine-primed BALB/c mice. The protocol was approved by Institutional Animal Care and Use Committee (IACUC) of the Biological Resource Center, A*STAR, Singapore (Protocol Number: 110694). In order to generate rabbit anti-H5N1-NS1 polyclonal antibody, GST-fusion NS1 protein was purified using the method as previously described^28^. New Zealand white rabbits were then immunized with this protein and bled as previously described^40^. The protocol was approved by IACUC of the Biological Resource Center, A*STAR, Singapore (Protocol Number: 110693). All the animal procedures were performed in strict compliance with the recommendations of the NACLAR guideline in Singapore. All efforts were made to minimize the suffering and euthanasia was performed using carbon dioxide.

### Protein expression and purification

NS1(RBD) (residues 1–73) of A/chicken/Hatay/2004(H5N1) was PCR amplified from a full-length NS1 gene (Accession NO.: CAJ01906.1) and the NS1(RBD) (residues 1–70) of A/Puerto Rico/8/1934(H1N1) was PCR amplified from a full-length NS1 gene (Accession NO.: ABD77680.1). The expression constructs were generated by inserting PCR product into pET SUMO expression vector (Invitrogen). NS1 mutants expression construct was generated by overlap PCR as described previously^41^. All proteins were expressed and purified as previously described^29^. The purified proteins were dialyzed against dialysis buffer (20 mM Tris-HCl, pH 7.4, 100 mM NaCl) and concentrated to 3 mg/ml in a Centrprep-10 (Amicon) for subsequent assays.

### Crystallization of 2H6-Fab

MAb 2H6 was purified from the ascites by using affinity chromatography and 2H6-Fab was obtained by papain cleavage as described previously^29^. The purified 2H6-Fab was dialyzed against dialysis buffer and concentrated to 10 mg/ml for subsequent crystallization. Crystallizations were performed with the hanging drop vapor diffusion method at 20 °C by mixing 1 μl of 2H6-Fab with 1 μl of reservoir solution and the mixture was equilibrated against 600 μl of reservoir solution. Crystals of 2H6-Fab were grown against crystallization buffer containing 25% PEG 3350, 0.2 M NaCl and 0.1 M Bis-Tris, pH 6.5. These crystals grew to a maximum size of 0.3 mm × 0.1 mm × 0.1 mm over the course of 10 days. Single crystals were obtained by dissecting from multiple crystals. Crystals were flash frozen (100 K) in the above reservoir solution supplemented with 30% glycerol. A total of 360 frames of a native data set with 1 oscillation at 1.5418 Å wavelength were collected for 2H6-Fab. All data sets were processed by HKL2000. Most of the crystals diffracted rather weak and the scaled data sets were anisotropic with strong ice rings and high mosaicities. Nevertheless, one of the data sets displaying weak ice ring was able to scale to 2.4 Å at the mosaicity of 1.8°. This data was used for structure determination and refinement with the statistic table listed as Table 1.

### Generation of 2H6-Fab and NS1(RBD) complex model by HADDOCK

VH-CDRs and VL-CDRs of 2H6-Fab were predicted using the online IgBlast tool (http://www.ncbi.nlm.nih.gov/igblast/). As the three-dimensional structure of NS1(RBD) of A/chicken/Hatay/2004(H5N1) has not been solved, the structure of NS1(RBD) of A/crow/Kyoto/T1/2004(H5N1) (PDB ID: 2Z0A) was used instead. As shown in Fig. 1A, there are 3 amino acid differences between the NS1(RBD) of these two strains but the sequence of helix α2 in the NS1(RBD) is 100% identical. Mutagenesis data from comparative ELISA was used to generate a series of models for the complex between 2H6-Fab and NS1(RBD) by using version 2.2 of HADDOCK webserver^32^,^42^. Along with the available individual structure, HADDOCK utilizes the experimentally derived data to predict the complex structure. To achieve this, NS1(RBD) was docked to 2H6-Fab using the Easy Interface of HADDOCK webserver, where both residues N48 and T49 of NS1(RBD) and amino acids in the CDRs of 2H6-Fab were defined as active residues involved in the interaction. In this docking experiment, only the variable domains of 2H6-Fab were used.

### Enzyme-linked immunosorbent assay (ELISA)

ELISA was performed as described previously^29^. Briefly, a synthetic peptide corresponding to residues 30–53 in H5N1-NS1 (APFLDRLRRDQKSLRGRGNTLGLD, chemically synthesized by GL Biochem) or purified WT and mutant NS1(RBD) proteins were serially diluted into 0.05 M carbonate–bicarbonate buffer, pH 9.6. A 29-mer peptide corresponding to a fragment of the hepatitis C virus core protein (RPSWGPIDPRRRSKNLGKVIDTLTCGFAP, chemically synthesized by Genway Biotech) was used as a negative control. Proteins or peptides (50 μl) were then coated onto 96-well ELISA plates (Nunc) overnight at 4 °C. The wells were blocked in 5% milk in PBS with 0.1% Tween 20 (PBST) for 1 h at 37 °C followed by addition of 100 μL of mAb 2H6 as primary antibody to each well and incubated at 37 °C for 2–3 h. The wells were then washed in PBST followed by the addition of goat anti-mouse horse-radish peroxidase (HRP)-conjugated antibody (Pierce) as secondary antibody and incubated at 37 °C for 1 h. Tetramethylbenzidine substrate (Pierce) was added and reaction was stopped using 0.2 M sulfuric acid. Absorbance at 450 nm was recorded using an absorbance reader (Tecan Infinite M200).

### Generation of recombinant Influenza/A/Puerto Rico/8/1934 virus from cloned DNA

Recombinant viruses were generated with pHW2000 reverse genetic system as described previously^43^. Residue 48 in NS1 was changed from S to A or N by PCR mutagenesis and the resulting DNA were inserted into pHW2000 vector. This plasmid was co-transfected with another seven pHW2000 plasmids containing the other PR8 genomic DNAs into 293T cells. 2 days post transfection, culture supernatant was collected and used to infect MDCK cells. When cytopathic effect (CPE) was visibly detected, culture supernatant was collected and used to infect naïve MDCK cells. Individual plaque was then amplified and viral titer was determined by plaque assay.

### Virus Infection and Western blot analysis

80% confluent 293T cells were infected with 2 MOI of WT or mutant viruses at 37 °C for 1 h. The medium was discarded and cells were rinsed with PBS. Cell lysates were harvested at 12 and 24 h.p.i. in RIPA buffer (50 mM Tris-HCl pH 8.0, 150 mM NaCl, 0.5% NP40, 0.5% sodium deoxycholate, 0.005% SDS). Then, 25 μg of total lysate were resolved using electrophoresis on an SDS–polyacrylamide gel and transferred to a nitrocellulose membrane (Bio-Rad). Antibodies against GAPDH (Santa Cruz), NP (Millipore), and M1 (as described previously)^29^ were used. Mab 2H6 and rabbit anti-H5N1-NS1 polyclonal antibody (as described above) were also used. After washing, the membrane was incubated with a HRP-conjugated secondary antibody (Pierce) at room temperature for 1 h. The membranes were then washed and detected with enhanced chemiluminescence substrate (Pierce) using ChemiDoc™ MP Imaging System (Bio-Rad).

### Multiple-cycle growth kinetics of recombinant virus

Plaque assay was applied to determine the growth kinetics of rgPR8-NS1-WT, rgPR8-NS1-S48A and rgPR8-NS1-S48N recombinant viruses. 90% confluent monolayers of A549 cells were rinsed with PBS and subsequently adsorbed with 0.01 MOI of WT and mutant viruses respectively for 1 h at 37 °C. The medium was discarded and the cells were rinsed using PBS and cultured in MEM without serum at 37 °C. Supernatant containing virus was collected at 12, 24, 36, 48 and 60 h.p.i. respectively and subjected to plaque assay to determine viral titer.

### Plaque assay in MDCK cells

90% confluent MDCK cells were adsorbed with serially diluted supernatants containing viruses for 1 h at 37 °C. The medium was discarded and the cells were rinsed using PBS. The cells were overlaid with 2 ml of DMEM supplemented by 0.3% agar and 2 μg/ml TPCK-trypsin (Thermo Scientific). After incubation at 37 °C for 2 days, the cells were fixed using 10% formalin for 1 h and stained using 0.1% crystal violet solution.

### Delivery of 1A9-Fab and 2H6-Fab into A549 cells

A549 cells were cultured in 12-well plate. The 1A9-Fab and 2H6-Fab were then transfected into 80% confluent cells by using Lipodin-Ab reagent (Abbiotec) according to manufacturer’s protocol with slight modification. Briefly, 40 μl of Fab solution (4 μg) was mixed thoroughly with 4 μl Lipodin-Ab solution and incubated for 15 min at room temperature. 100 μl of serum-free MEM medium was added to Fab/Lipodin-Ab solution and then immediately added to the cells. The cells were washed with PBS once before the Fab/Lipodin-Ab solution was added. The cells were incubated at 37 °C in 5% CO_2_ for 12 h, washed once with PBS and subjected to viral infection and western blot analysis as described above.

### Cell viability assay

Cell viability was determined using WST-1 reagent (Roche) according to manufacturer’s protocol. Briefly, 10 μl of WST-1 reagent was diluted in the 90 μl of culture media and added to A549 cells cultured in a transparent 96-well microplate and incubated for 1 h, followed by measuring absorbance at 440 nm.

### Immunofluorescence assay

A549 cells grown on coverslip were transfected with Fab as described above. Approximately 12 h after transfection, the medium was aspirated, washed with PBS once and replaced with serum-free MEM medium and incubated at 37 °C in 5% CO_2_ for another 24 h. The cells were then washed with PBS once and fixed with 4% paraformaldehyde for 10 min and permeabilized with 0.1% TritonX-100 for 10 min, followed by blocking with 1% BSA in PBS for 30 min. The cells were incubated with Alexa Fluor 488-conjugated goat anti-mouse IgG antibody (Invitrogen) for 1 h. After washing, cells were stained with DAPI before mounting. Images were captured using an Olympus FluoView FV1000 laser-scanning confocal microscope.

### AlphaScreen-based dsRNA binding inhibition assay

A 21nt-siRNA previously reported to form complex with NS1(RBD)^34^ was purchased from Thermo Scientific Dharmacon (Dharmacon, Lafayette, CO). The siRNA was biotinylated at the 5′ end of the sense strand and the siRNA sequences were as follows:

5′-Biotin-AGACACCAUUAUGCUGUCUUU-3′ (sense) and 5′-AGACAGCAUAAUGGUGUCUUU-3′ (antisense). The lyophilized siRNAs were reconstituted in RNase-free water to a final concentration of 50 nM. To express GST-tagged NS1(RBD) of A/chicken/Hatay/2004(H5N1) was PCR amplified from a full-length NS1 gene (Accession NO.: CAJ01906.1) and cloned into pGEX-6P-1 vector (GE healthcare). The expression and purification were performed as described previously^28^. The purified GST-fusion protein was dialysed against PBS and the final concentration was determined using the Coomassie Plus protein assay reagent (Thermo Scientific).

*In vitro* RNA binding inhibition assay was carried out in 384-well ProxiPlate by using the AlphaScreen anti-GST kit (PerkinElmer). In the first experiment, 5 μl of 50 nM GST-tagged proteins were mixed with same volume of serially diluted mAb 2H6 and incubated at room temperature for 1 h. Then, 5 μl of 50 nM biotinylated 21nt-siRNA was added into the binding mixture and incubated at room temperature for 1 h before the addition of 10 μl of detection mixture containing 0.1 μl of anti-GST (Glutathione S-Transferase) acceptor beads and 0.1 μl of streptavidin-coated donor beads (PerkinElmer). After another incubation at room temperature for 1 h, luminescent signal was measured using an EnSpire multimode plate reader (PerkinElmer)^35^. In the second experiment, 5 μl serially diluted mAb 2H6 was mixed with 5 μl of 50 nM GST-tagged proteins and 5 μl of 50 nM biotinylated 21nt-siRNA simultaneously. After incubation at room temperature for 1 h, detection mixture was added and measurement was made similarly.

### Statistical analysis

Two-tailed Student’s t test was applied to evaluate the statistical significance of differences measured from the data sets obtained in 3 independent experiments. *p* < 0.05 was considered statistically significant.

## Additional Information

**Accession codes:** The coordinate has been deposited in the PDB under the accession code 5B4M.

**How to cite this article**: Wu, J. *et al.* Biochemical and structural characterization of the interface mediating interaction between the influenza A virus non-structural protein-1 and a monoclonal antibody. *Sci. Rep.* **6**, 33382; doi: 10.1038/srep33382 (2016).

## Supplementary Material

Supplementary Information

## Figures and Tables

**Table 1:** Data collection and refinement statistics for 2H6-Fab structure.

Data collection	
Space group	P2 (1)
PDBID	5B4M
Cell dimensions	
*a*, *b*, *c* (Å)	51.59, 90.94, 81.44
*Β* (°)	92.2
Wavelength (Å)	1.5418
Resolution (Å)^a^	50~2.4 (2.44~2.40)
Rsym (%)	7.0 (44.5)
*I*/σ(*I*)	16.1 (2.2)
Completeness (%)^a^	99.7 (97.7)
Redundancy	3.5 (3.4)
Search model	4Q9Q
Refinement	
Resolution Range (Å)	50~2.40 (2.47~2.40)
No. reflections	27,507
Rwork (Rfree) (%)	28.5/33.8 (34.6/42.2)
No. atoms	
Protein	6,738
Water	22
B-factors (Å^2^)	
Protein	39.93
Water	29.85
R.m.s. deviations	
Bond lengths (Å)	0.012
Bond angles (°)	1.561
% favored (disallowed) in Ramachandran plot	86.8 (0)

^a^Values for the highest-resolution shell are in parentheses.

**Table 2:** Comparison of amino acid signature at residue 48 in the NS1 proteins of human and avian influenza A viruses.

Subtype	Host	Year Range	Sequence No.	Frequency (%)		
				N	S	Others
H5N1	Avian	1959–2015	2267	86.7	13.2	0.1
H1N1	Human	1918–2008	1336	7.2	92.4	0.4
pdmH1N1	Human	2009–2015	5998	99.7	0.1	0.2
H3N2	Human	1968–2008	3255	90.8	7.8	1.4
		2009–2015	3888	99.4	0.2	0.4
