# Supplementary material for: Biochemical and structural characterization of the interface mediating interaction between the influenza A virus non-structural protein-1 and a monoclonal antibody
Source: Sci Rep. 2016 Sep 16;6:33382. doi: 10.1038/srep33382 (PMC5025888; doi:10.1038/srep33382)

Supplementary figures

Biochemical and structural characterization of the interface mediating interaction between the influenza A virus non-structural protein-1 and a monoclonal antibody

Jianping Wu[1], Chee-Keng Mok[1], Vincent Tak Kwong Chow[1], Y. Adam Yuan[2,3] and Yee-Joo Tan[1,4,*]

[1]Department of Microbiology and Immunology, Yong Loo Lin School of Medicine, National University Health System (NUHS), National University of Singapore, [2]Department of Biological Sciences, Faculty of Science, National University of Singapore, [3]National University of Singapore (Suzhou) Research Institute, Suzhou Industrial Park, Jiangsu 215123, China, [4]Institute of Molecular and Cell Biology, A*STAR (Agency for Science, Technology and Research), Singapore.

*Correspondence author (email: yee_joo_tan@nuhs.edu.sg)

Legends for supplementary figures

Figure S1. Binding profile of mAb 2H6 to WT and mutant H5N1-NS1(RBD) by ELISA. Comparative ELISA was performed to determine the residues in NS1 that are critical for its interaction with mAb 2H6. Serially diluted proteins were coated onto the ELISA plate and probed with 5 µg/ml of mAb 2H6. All experiments were performed in triplicates, and the average values with SD were plotted.

Figure S2. HADDOCK-derived models of 2H6-Fab and H5N1-NS1(RBD) complex. Model 2 of cluster 1 (A and B) and model 3 of cluster 3 (C and D) were generated by HADDOCK webserver. (A and C) Ribbon diagram of HADDOCK-derived models displayed complex structure of H5N1-NS1(RBD) coloured in cyan, 2H6-Fab heavy chain coloured in blue and light chain coloured in brown. VH-CDR1, CDR2 and CDR3 of 2H6-Fab were coloured in red, yellow and orange respectively, and VL-CDR1, CDR2 and CDR3 were coloured in light blue, wheat and purple respectively. (B and D) Focused view of the binding interface displaying the residues involved in the interaction between antigen and antibody. Residues of H5N1-NS1(RBD) and 2H6-Fab predicted critical for the binding are shown in the stick mode and coloured in red and black respectively. Probable hydrogen bonds are indicated by dashed lines. All images of the models were generated with PyMOL.

Figure S1

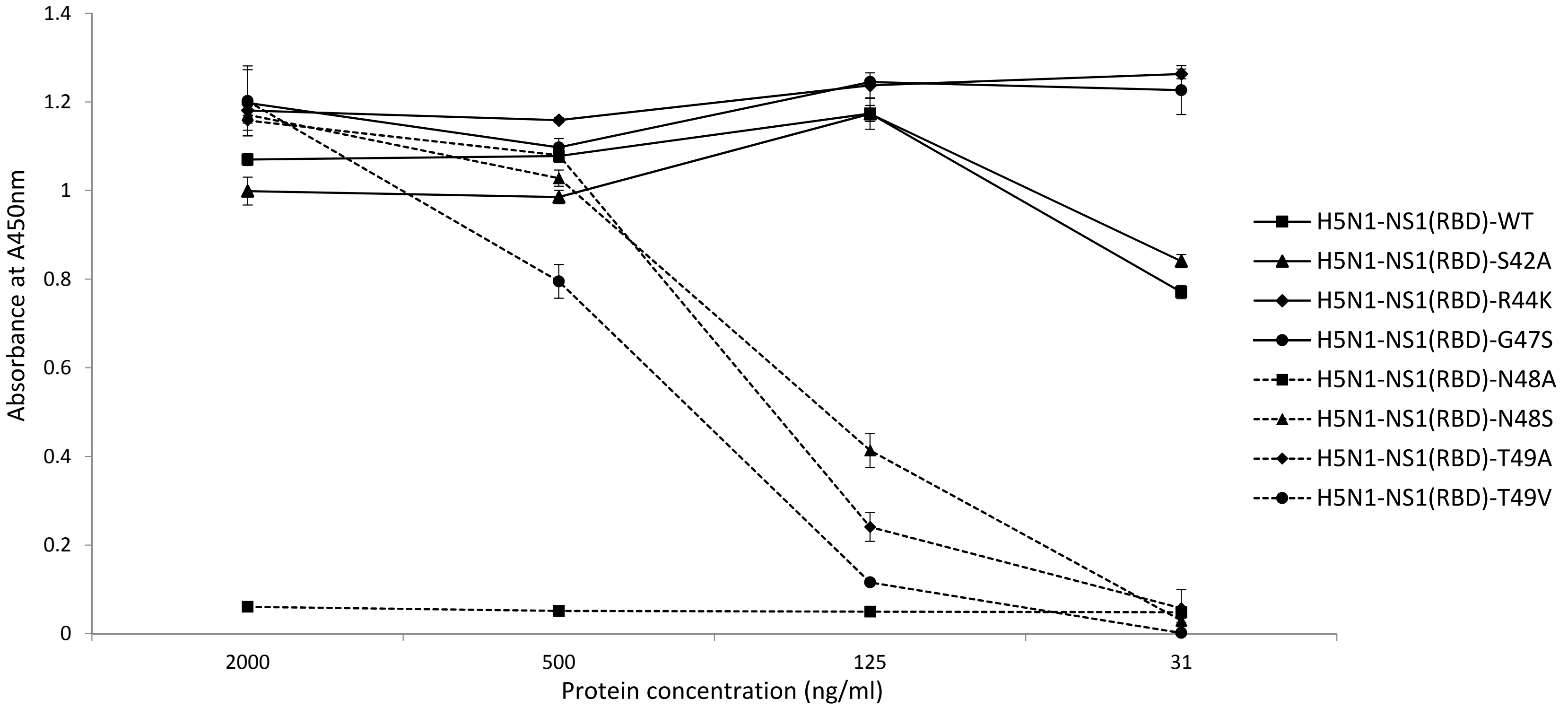

1.4
1.2
1
0.8
0.6
0.4
0.2
0
Absorbance at A450nm
2000
500
125
31
Protein concentration (ng/ml)
H5N1-NS1(RBD)-WT
H5N1-NS1(RBD)-S42A
H5N1-NS1(RBD)-R44K
H5N1-NS1(RBD)-G47S
H5N1-NS1(RBD)-N48A
H5N1-NS1(RBD)-N48S
H5N1-NS1(RBD)-T49A
H5N1-NS1(RBD)-T49V

Figure S2

A

B

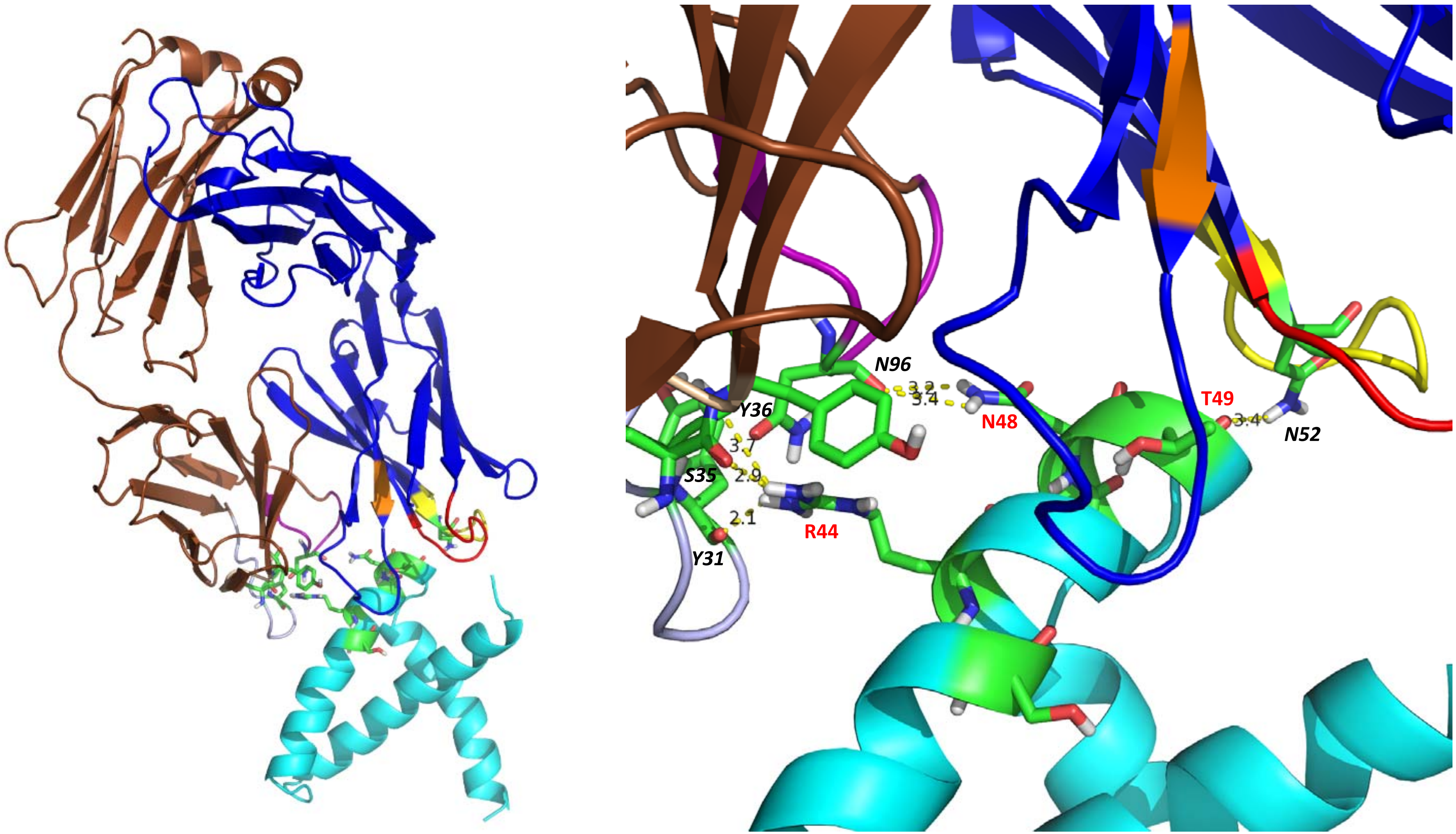

Figure S2

C

D

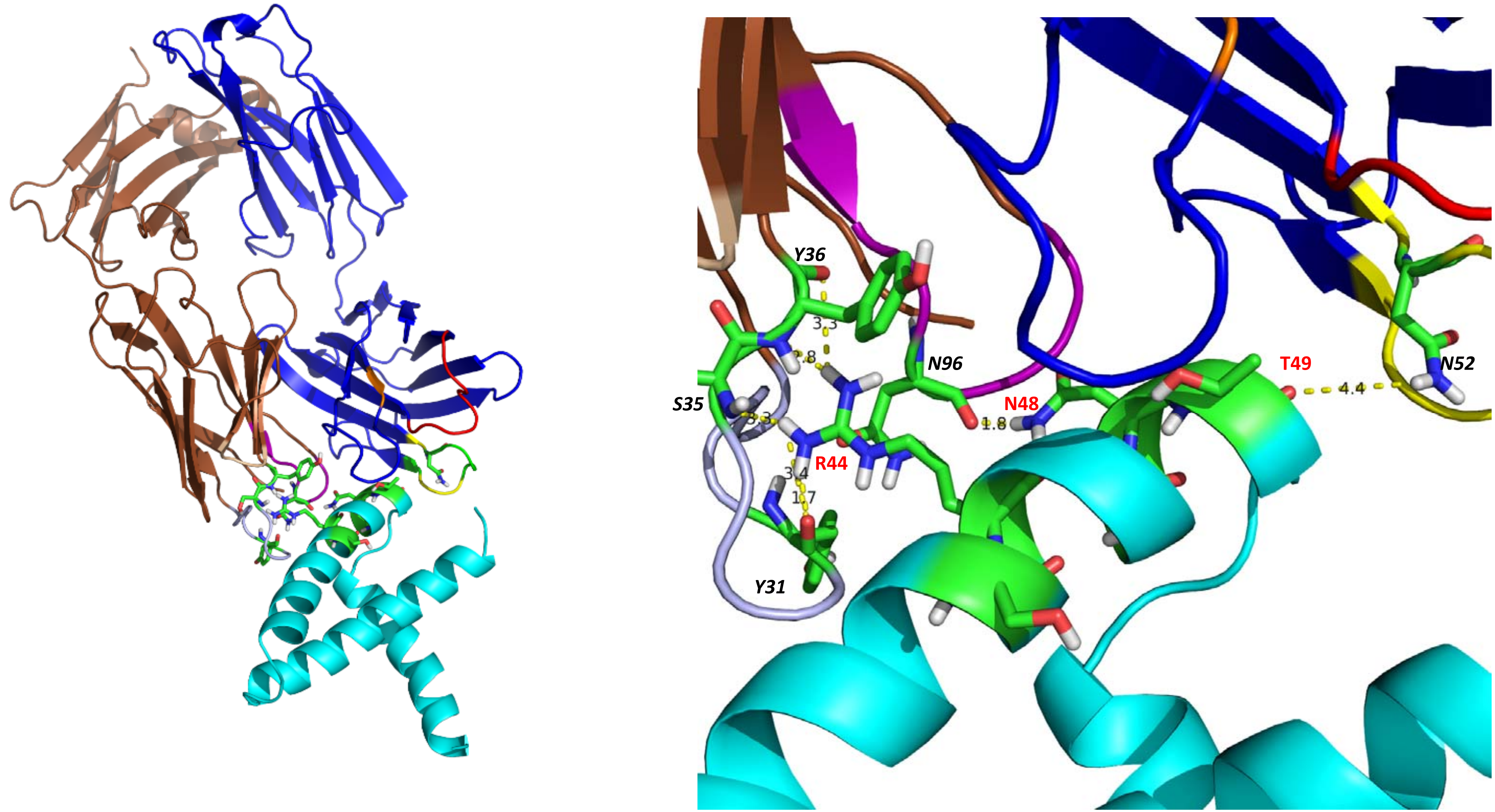

Supplement: Supplementary Information [file srep33382-s1.pdf]
